# 0.5 Gy confers resistance to a subsequent high dose of γ-rays by modulating HO-1/Nrf2 and apoptosis pathways

**DOI:** 10.1038/s41598-025-91667-9

**Published:** 2025-03-17

**Authors:** Yasser F. Ali, Ibrahim M. Hassan, Hussein M. Abdelhafez, Omar S. Desouky

**Affiliations:** 1 Biophysics lab, Physics Department, Faculty of Science, Al-Azhar University, Nasr city, Cairo, 11884 Egypt; 2 Radiation Physics Department, National Center for Radiation Research and Technology, Egyptian Atomic Energy Authority, Cairo, Egypt

**Keywords:** Adaptive response, 0.5 Gy, HMOX1, Low dose, Apoptosis pathways, Biological physics, DNA damage and repair

## Abstract

Ionizing radiation, from the DNA centric view, elicits biological effects and health consequences solely through energy deposition events in the cell nucleus. At higher radiation doses, this is likely true; however, at low doses, non-targeted effects, a subcategory of which is the adaptive response, tend to dominate. Controversies exist over the definition of low dose. From a radiation therapy view, it is defined as 0.5–0.7 Gy. Therefore, we investigated the effects of exposure to ionizing radiation with or without a 0.5 Gy priming dose. Techniques including comet assay, flow cytometry, fluorescence microscopy, and real-time quantitative PCR were employed. In normal lung fibroblasts (WI-38), there was a statistically significant difference in mean normalized tail moments when comparing treatment with the challenge dose alone to treatment with a 0.5 Gy priming dose prior to the challenge dose (*P* < 0.05). Moreover, pretreatment with a 0.5 Gy priming dose reduced G1 phase cell cycle arrest and cell death—either through apoptosis or mitotic catastrophe—induced by the subsequent 2 Gy exposure. Similarly, A549 Cells pre-exposed to a 0.5 Gy priming dose before a 2 Gy exposure showed a lower percentage of apoptosis than those exposed to the 2 Gy alone. Mechanistically, cells responded to a priming 0.5 Gy by increasing the expression of HMOX1, SOD, and Bcl2 while decreasing of IL-1β and TNF-α. In conclusion, 0.5 Gy induces an adaptive response in lung normal and cancer cell against subsequent high doses of γ-rays. Modulation of the HO-1/Nrf2 and apoptosis pathways underlie the resistance observed in primed cells.

## Introduction

One way to prime the body before a larger dose of radiation is to use low-dose ionizing radiation (LDIR), which is defined as those that do not exceed 100 mGy of low-LET radiation according to the classification provided by UNSCEAR^1^ In the study by Ito et al.^2^, a mid-lethal challenging dose of X-ray (5.9 Gy) applied to 8-week-old C57BL/6 mice twenty-four hours after a 50 mGy priming dose. They reported an increase in the survival rate of mice compared to irradiated control. The pKZ1 transgenic mouse model has been previously used to measure dose response for DNA inversions in spleen and prostate for a wide dose range. Curiously, dose as low as 0.001 mGy completely protected against chromosomal inversions induced by a single high dose of 1 Gy, and, also, against a proportion of spontaneously induced inversions^3^. In cancer-prone animals with a partial TP53 function, a single whole-body dose of 10 mGy does increases cancer latency and effectively restores a portion of the life that would have been lost due to either spontaneous or radiation-induced cancer in the absence of the low dose^4^. Increasing the priming dose to 100 mGy has imaginarily eliminated the protective effects. In other words, 100 mGy exceeded the upper dose threshold for induction of adaptive protection. Conversely, thymocyte apoptosis induced by a challenge dose of 2 Gy was significantly decreased when the mice were preirradiated with a 100 mGy priming dose 6 h prior to the challenge dose^5^, hence upper threshold dose appear to be higher than previously considered. An epidemiological study shows that no excess cancers have been found at doses below 200 mGy^6^, leading to the definition of low dose as 200 mGy or less^7^. However, from a radiation therapy view, doses in the range of 0.5–0.7 Gy are defined as a low^8^. Exposure to an X-ray dose of 500 mGy did not render human lymphocytes refractory to chromatid deletions caused by a challenge dose of 1 Gy^9^. Similar observations were reported by Broome et al.^10^ using normal human fibroblasts. By contrast, Wistar rats have recently been found to express lower MDA and NO levels after total-body irradiation with a dose of 5 Gy if pre-treated several hours earlier with low dose of the order of 0.5 Gy^11^. Conflicting findings from both sides of the radiation hormesis argument indicate that the exact effects at so-called “low dose” in radiation therapy view are not known. In the current study we investigated whether the pre-exposure to 0.5 Gy as a priming dose can protect from the damaging effects of subsequent high one (2 Gy). A variety of endpoints at different times following the high dose exposure were measured: mitotic catastrophe after 3 days; DNA damage, cell cycle redistribution, and apoptosis at earlier time points, 1 to 24 h post-exposure. Whether adaptive response could be induced in tumor cells by LDIR has likewise remained elusive. Studies have revealed that tumor cells are resistant to LDIR-induced adaptive response^12^ or showed a distinct pattern from normal cells^13^. Cultures pre-exposed to 75 mGy followed by 4 Gy at a 12-hour interval had fewer surviving cells than those exposed to 4 Gy alone, indicating that the predose significantly enhanced the tumor-killing effect of the subsequent high one^13^. This was confirmed in vivo using S180-bearing mice^14^. Therefore, the present study was also aimed at determining the ability of 0.5 Gy to induce an adaptive response in A549 cells to a subsequent challenging dose of 2 Gy.

## Materials and methods

### Cell line and cell culture

Human cancer-derived cell A549 and normal fibroblast called human embryonic fibroblast, lung-derived cell line (WI-38) were obtained from VACSERA -tissue culture unit (Giza, Egypt). Cells were cultured in Dulbecco’s modified Eagle’s medium (DMEM, Gibco) supplemented with 1% antibiotic-antimycotic solution (10,000 units/ml of penicillin, 10,000 µg/ml of streptomycin, and 25 µg/ml of amphotericin B) and 10% fetal bovine serum (FBS, Gibco). Cells were grown in 25–75 cm culture flasks and maintained in a humidified 37 °C incubator with 5% CO_2_.

### Irradiation

Cells were exposed to γ-radiation doses of 0–0.5 Gy 24 h before being exposed to a challenge dose of 2 Gy. The irradiation procedure was performed at the Atomic Energy Authority, Cairo, Egypt, using the Canadian gamma cell-40 (^137^Cs) at a dose rate of 0.333 Gy/min. Cells were irradiated under aerobic conditions at room temperature and returned to the incubator between priming and challenge dose exposures.

### Comet assay

Cells were harvested 1 h post exposure to 2 Gy γ-irradiation and then subjected to the comet assay in alkaline solution using the Comet Assay Kit (Abcam, ab238544) according to the manufacturer’s protocol. Briefly, ∼ 10^5^ cells were combined with Comet Agarose at a 1:10 ratio and immediately transferred onto the slide glasses covered with a comet agarose base layer. Slides were stored at 4 °C in the dark for 15 min before being immersed in lysis buffer for 60 min. This was then followed by immersion in an alkaline unwinding solution for 30 min. Slides were inserted into the electrophoresis chamber, immersed in the buffer, and an electric potential of 1 V/cm was applied for 20 min. After electrophoresis, the slides were rinsed three times (each for 5 min) with deionized water, followed by a single wash with 70% ice-cold ethanol, and then dried at room temperature. Once dry, 100 µl/well of 1× Vista Green DNA Dye was added, and microscopy images were taken using the Labomed LX400 fluorescent microscope. Comet tail moments were analyzed using the CometScore™ software from TriTek Corporation. Cells analyzed were categorized into three DNA damage groups based on their tail moment values. Cells with a tail moment less than 2 were classified as undamaged (comet type 1); cells with a tail moment of 2 or higher were considered damaged (comet types 2, 3, 4, and 5). Among damaged cells, those with tail moment values exceeding 30 were designated as apoptotic (comet type 5b).

### Apoptosis analysis

Apoptosis was quantitated using the FITC Annexin V/Dead Cell Apoptosis Kit (Invitrogen, Cat# V13242) according to the manufacturer’s instructions. Briefly, after 24 h from 2 Gy exposure, cells were harvested by trypsinization and washed with cold PBS. After centrifugation (1000 rpm, 5 min), cell pellets were resuspended in 100 µL 1× annexin binding buffer. Subsequently, 5 µL of FITC Annexin V and 1 µL of a propidium iodide solution (100 µg/mL) were added. After a 15 min incubation at room temperature, the mixture was diluted by adding 400 µL of 1× annexin binding buffer. Acquisition and analysis were performed on a Navios Flow Cytometer (Beckman coulter life science, USA) using the Navios software.

### Cell cycle analysis

Cell cycle status was determined using Vybrant™ DyeCycle™ Violet Stain (Thermo Fisher Scientific, Cat# V35003) according to the manufacturer’s protocols. Cells were collected 24 h after 2 Gy irradiation by trypsinization, fixed in ice-cold methanol and stained with the DNA-selective stain Vybrant DyeCycle Violet. Eventually, the stained cells were analyzed on a Beckman Coulter Navios flow cytometer with excitation at 405 nm and emission measured at 440 nm.

### Mitotic catastrophe

Cells were seeded on sterile coverslips placed in four-well plates. After overnight attachment, cells were irradiated as indicated. Subsequently, Cells were fixed 72 h after the final radiation dose (2 Gy) using a 4% paraformaldehyde solution in 1× PBS for 20 min at room temperature. After one PBS wash, coverslips were mounted, cell side down, onto labelled microscope slides using ProLong™ Gold Antifade Mountant with DAPI (Thermo Fisher, Cat# P36935). Nuclear morphology was examined under a fluorescence microscope (Labomed LX400). Cells containing nuclei with two or more distinct lobes or aberrant nucleus morphology were scored as undergoing mitotic catastrophe^15^.

### RNA extraction and quantitative real-time PCR (qRT-PCR)

Total RNA was extracted from cells 24 h after exposure to 0.5–2 Gy using the Qiagen RNeasy Mini Kit (Cat# 74104) according to the manufacturer’s protocol, and subsequently reverse transcribed to cDNA with the QuantiTect Reverse Transcription kit (Qiagen, Cat# 205310). Thermal cycling parameters for the reverse transcription reactions were: 15 min at 42 °C, then 3 min at 95 °C, followed by holding on ice. qRT-PCR was performed on a 5 plex Rotor-Gene PCR Analyzer (Qiagen) using SYBR Green PCR Master Mix (QuantiTect SYBR green PCR kit, Cat# 204141) and the following QuantiTect primers: HMOX1 (QT00092645), BCL2 (QT00025011), CDK6 (QT00019985), and WEE1 (QT00038199). Gene Expression was normalized to the housekeeping gene, ACTB (QT000954231), and relative gene expression was determined using the 2^− ΔΔCt^ method.

### Statistical analysis

All experiments were independently performed at least twice, and intergroup comparisons were analyzed using unpaired two-tailed Student’s t-tests. A P value below 0.05 was deemed statistically significant.

## Results

### The 0.5 Gy priming dose conferred WI-38 human lung fibroblasts a resistance to DNA damage induced after a large 2 Gy challenge dose

**Fig. 1:**
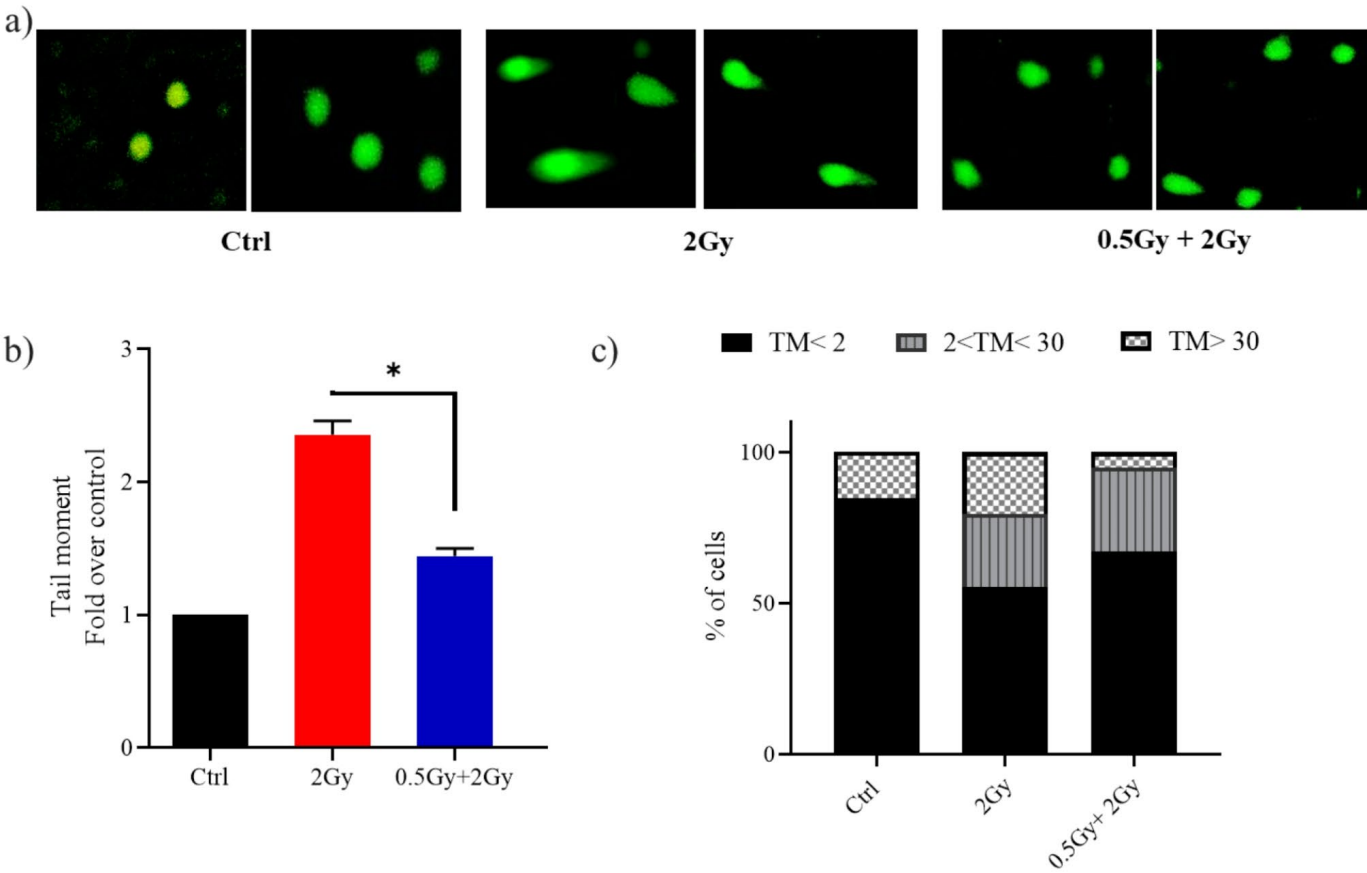
A priming dose of 0.5 Gy protected lung fibroblasts from DNA damage caused by a subsequent 2.0 Gy dose administered 24 h later. Representative images (**a**) and quantification (**b**) of tail moments in WI-38 cells at 1 h post 2 Gy irradiation. Thirty cells at least were analyzed in each group. Quantification is represented as values expressed as fold change relative to control (Ctrl). (**c**) Classification of DNA damage. A bar graph was used to show the distribution of cells among three DNA damage levels. Two independent experiments were conducted. The level of significance was represented as *P < 0.05, Student’s t-test.

DNA is the main cellular target damaged by exposure to ionizing radiation. Comet assay under alkaline condition helps in quantitative assessment of DNA damage. Tail moment, which is the product of tail DNA and tail length, was the parameter used to express the comet assay results. In WI-38 cell, treatment with a challenging dose of 2 Gy led to an increase in tail moments as compared to non-treated controls (Fig. 1). However, the magnitude of the increase in cells treated with both predose and challenging dose was less than that in those treated only with the challenging dose. These findings indicate that the cellular response to the challenging dose of radiation differs when cells are primed with 0.5 Gy.

Comets analyzed were classified by tail moment values to differentiate normal, damaged, and apoptotic cells, as described by Genghini et al.^16^. In cultures treated solely with the challenging dose, 45% of comets had tail moment values indicating damage, with 20% being apoptotic. In contrast, cultures treated with predose + challenging dose showed 33% of values indicating damage with 5% being apoptotic (Fig. 1c).

### The 0.5 Gy priming dose partially reversed the challenge dose-induced G0/G1 arrest and apoptosi

**Fig. 2:**
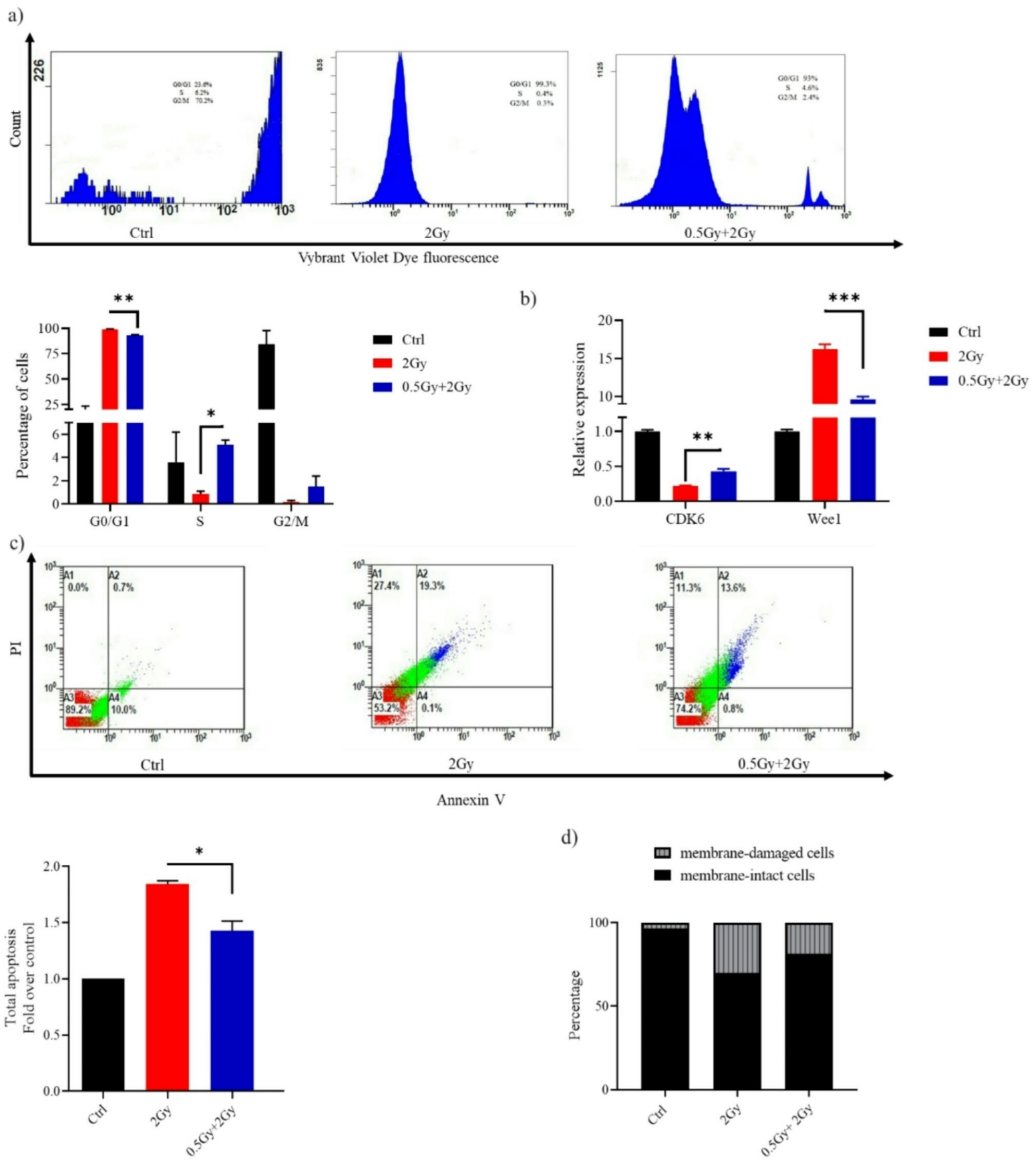
(**a**) Histograms obtained from flow cytometric analysis showing WI-38 cell cycle at 24 h after challenge with a dose of 2 Gy. (**b**) Reverse transcription-quantitative PCR results for the detection of CDK6 and Wee1 mRNA expression. (**c**) Representative flow cytometry dot plots of WI-38 cells after challenge with a dose of 2 Gy. At 24 h post-2 Gy IR, cells were harvested, stained with Annexin V/PI and analyzed using a flow cytometer. Apoptosis is shown as values expressed as fold change relative to control (Ctrl). (**d**) Bar graph depicting the percentage of membrane-intact and membrane-damaged cells following 2 Gy exposure, with and without a 0.5 Gy priming dose. Data are means ± SEM (*n* = 2); *P < 0.05, **P < 0.01 and ***P < 0.001 by Student’s t-test.

Following DNA damage, Cells either pause cell cycle progression until errors have been corrected or undergo apoptosis if the damage is massive and irreparable. In mock-irradiated controls, the cell-cycle phase distribution was 12% in G0/G1, 4% in S, and 84% in G2/M phase (top row, left panel of Fig. 2a). After a high dose exposure (2 Gy), WI-38 cells experienced a delay in moving out of G1 and into S phase, resulting in a cell cycle distribution of 99% in G0/G1, 0.8% in S, and 0.2% in G2/M (top row, middle panel of Fig. 2a). However, 0.5 Gy pretreatment of lung fibroblasts attenuated 2 Gy-induced G1 phase cell cycle arrest; released some cells from G1 arrest into S-phase (G0/G1:S: G2/M phases = 93%: 5%: 2%). Eukaryotic cells have about 20 cyclin-dependent kinases (CDK), but only CDK1-6 are related to the cell cycle. CDK4/6 specifically regulates cellular transition from the G1 phase to the S phase, where DNA synthesis occurs. Cells treated with the challenge dose alone showed lower *CDK6* levels and higher *Wee1* levels than those treated with the predose + challenge dose (Fig. 2b), indicating increased DNA damage induction. It is worth mentioning that Wee1 negatively regulates entry into mitosis (i.e., the G2/M transition).

The apoptotic rate in cells receiving the combination treatment was significantly lower than in those treated with the challenge dose alone (Fig. 2c). Flow cytometry analysis showed that 82% of cells treated with the predose + challenge dose retained membrane integrity, compared to 70% in the samples treated solely with the challenge dose (Fig. 2d).

### 0.5 Gy pretreatment markedly attenuated late-type cell death induced by subsequent 2 Gy exposure

**Fig. 3:**
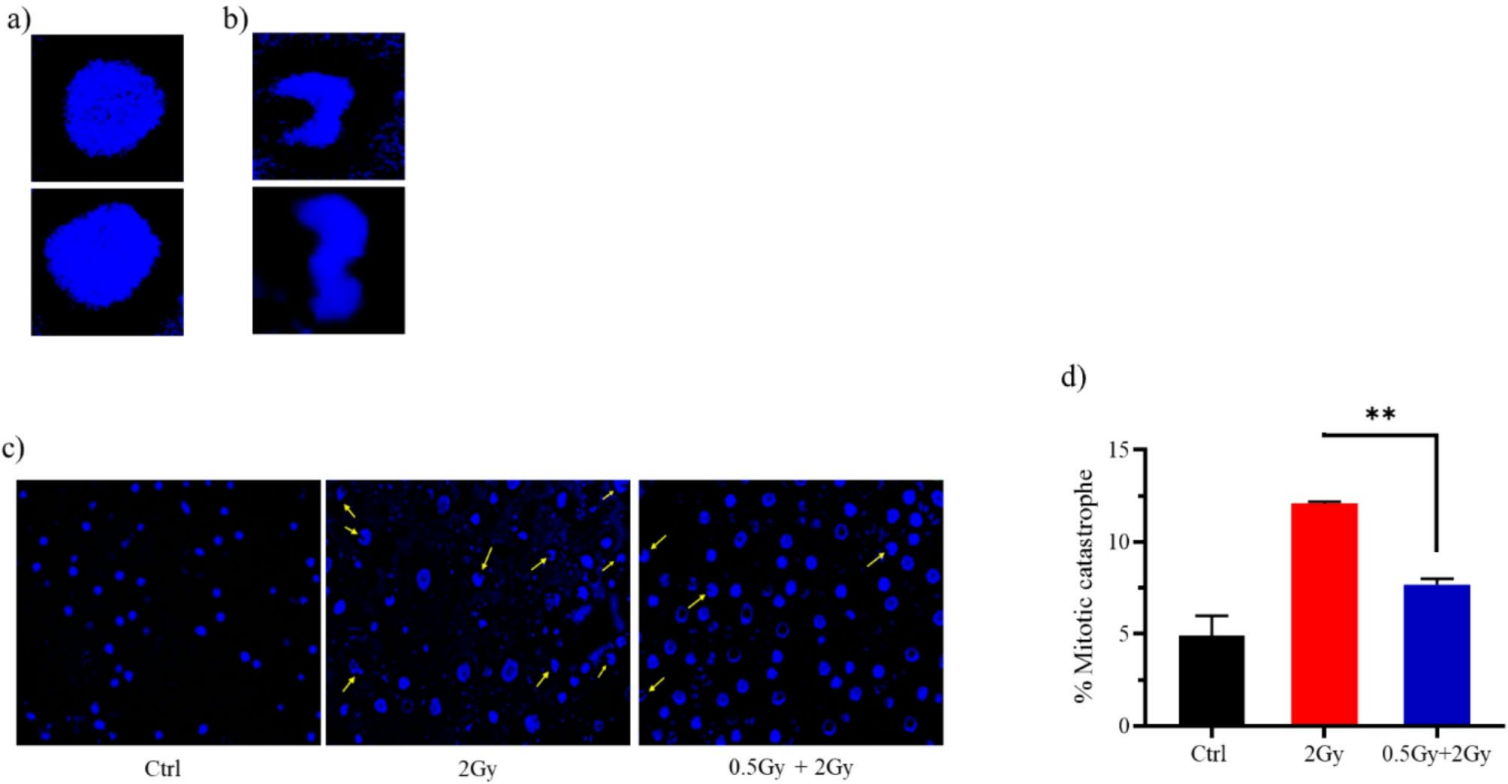
Representative images showing the nuclear morphology of (**a**) normal cells and (**b**) cells undergoing mitotic catastrophe. (**c**) Quantified data for mitotic catastrophe induced in cells treated with IR. 72 h after the final radiation dose (2 Gy), cells were fixed and stained with DAPI. Images of nuclei were acquired using a 40X lens. 100 nuclei were scored. Data are summary of two independent experiments and values are expressed in mean ± SEM. P values determined by two-sided Student’s t-test; **P < 0.01.

Mitotic catastrophe is a delayed cell death that typically occurs 2–6 days after irradiation. Cells undergoing mitotic catastrophe die during mitosis. Mechanistically, erroneous repair of radiation-induced DNA breaks leads to chromosome aberrations that subsequently prevent accurate segregation during mitosis, cause defects in mitotic exit, and ultimately result in cell death. Mitotic catastrophe is characterized morphologically by distinct nuclear changes, including a multilobed or aberrant appearance (Fig. 3a,b). DAPI staining revealed that 12% of cells treated solely with the challenging dose showed mitotic catastrophe, compared to only 7% in those treated with the predose + challenging dose (Fig. 3c,d).

### Molecular mechanisms underlying the hormetic effect of 0.5 Gy ionizing radiation in normal fibroblasts

**Fig. 4:**
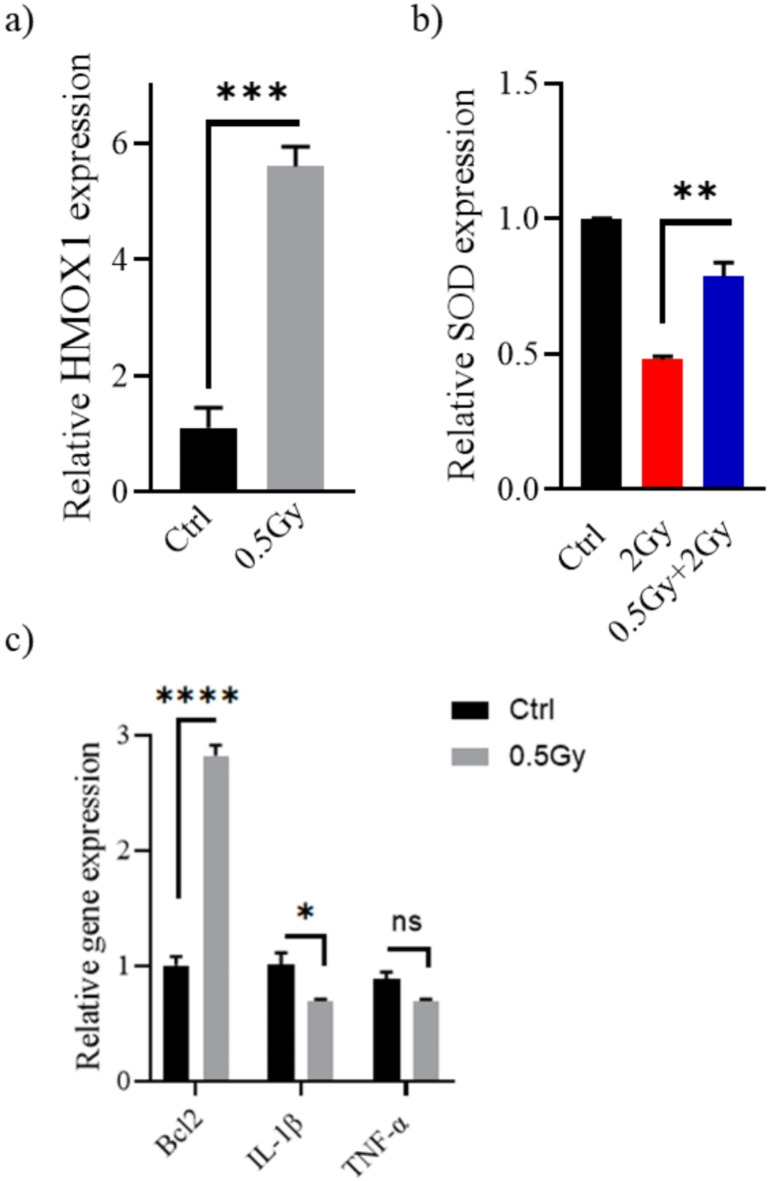
Changes in the (**a**) HMOX1, (**b**) SOD, (**c**) Bcl2, IL-1β, and TNF-α transcription levels in response to low dose irradiation. Twenty-four hours following exposure to radiation (0.5–2 Gy), WI-38 cells were harvested, and target genes expression relative to β-actin were determined by real-time PCR. *P < 0.05, **P < 0.01, ***P < 0.001, ****P < 0.0001 by Student’s t-test.

In WI-38, low-dose exposure of 0.5 Gy significantly increased *HMOX1* mRNA expression (Fig. 4a). Heme oxygenase 1 (HMOX1, commonly HO-1) plays a cytoprotective role. In response to stress, HO-1 moves to the nucleus and, by interacting with other proteins, regulates the transcription of antioxidant and cytoprotective genes. This is strongly supported by the observation that the cytotoxic effects of hydrogen peroxide and other pro-oxidant agents are exacerbated in cells that lack HO-1 (Hmox1^−/−^)^17^. High doses of ionizing radiation, on the other hand, reduce cell antioxidant concentrations^18^. Mechanistically, the balance between reactive oxygen species (ROS) production and antioxidant defense shifts in favor of ROS. Here, a dose of 2 Gy of gamma radiation clearly reduced levels of Superoxide dismutase (SOD), an important intracellular antioxidant (Fig. 4b). However, SOD levels in the 0.5 Gy + 2 Gy irradiated cells were significantly higher than in the 2 Gy irradiated cells (*P* < 0.01).

Apoptosis occurs through two major pathways: extrinsic and intrinsic pathways. Intrinsic apoptosis, activated by DNA damage, is primarily regulated by B-cell lymphoma 2 (Bcl-2) family proteins, which compose of both anti- and pro-apoptotic members. The anti-apoptotic subfamily includes Bcl-2, Bcl-XL, Bcl-W, Bcl-2-A1, and MCL1^19^. Exposure to 0.5 Gy increases Bcl2 levels in WI-38 (*P* < 0.001, Fig. 4c). On the other hand, the immune system regulates the extrinsic pathway of apoptosis. ROS generated from the higher challenge dose modulates cellular changes through non-nuclear interaction, increasing the transcription and subsequent release of pro-inflammatory cytokines such as tumor necrosis factor (TNF)-α and interleukin-1β (IL-1β). These cytokines induce cell death via the death receptor pathway^20,21^. Significantly lower concentrations of IL-1β were detected after predose treatment. Oppositely, TNFα levels were not significantly altered (*P* > 0.05). However, WI-38 cells showed a tendency to release a less TNFα after irradiation with 0.5 Gy (Fig. 4c). The reduced production of these pro-inflammatory cytokines and upregulation of Bcl2 may have given WI-38 cells resistance to apoptosis induced by a 2 Gy challenge dose.

### Pretreatment with 0.5 Gy protects lung cancer cells (A549) from apoptosis induced by subsequent 4 Gy exposure

**Fig. 5:**
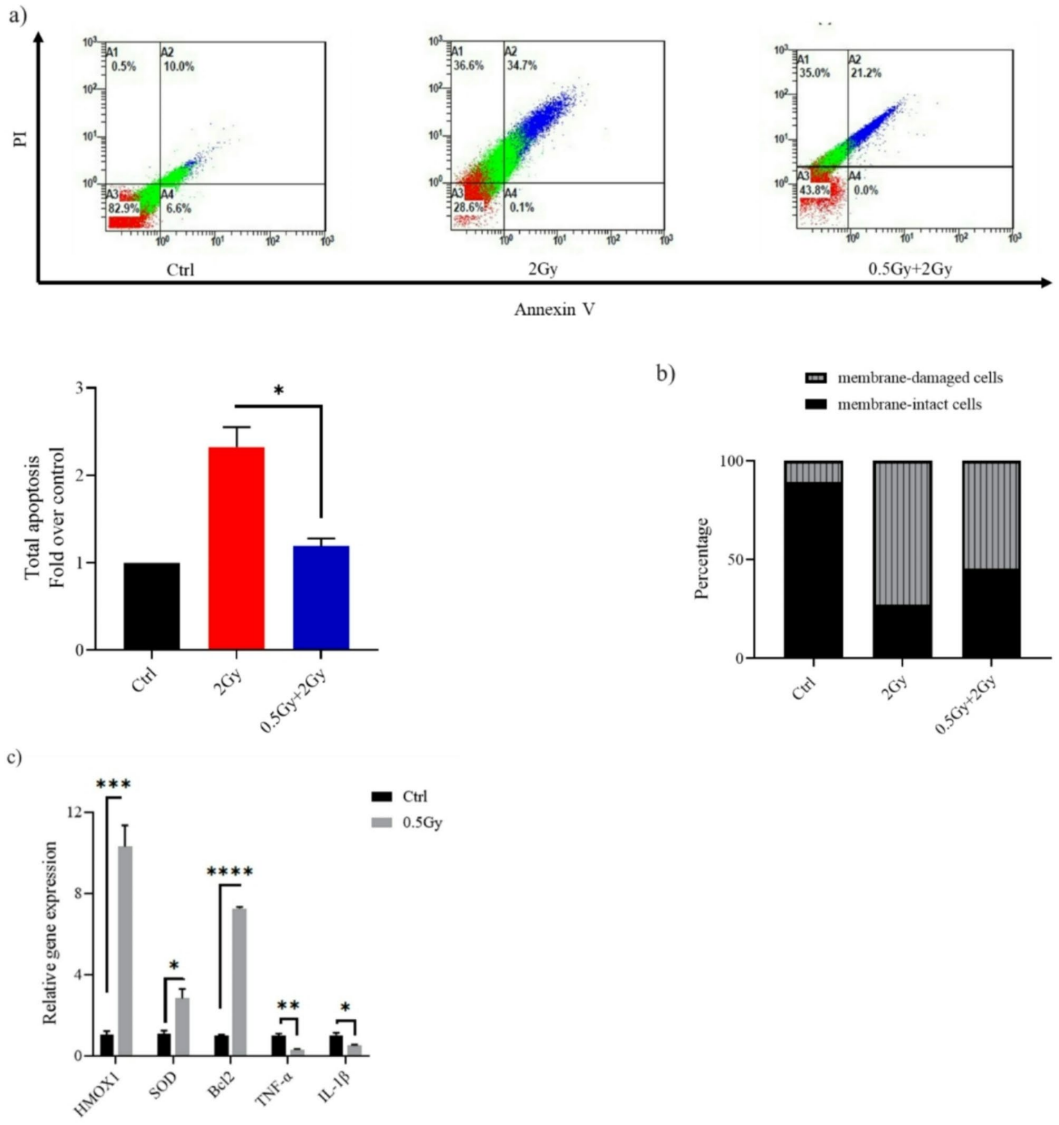
In A549, cell death induced after a large 2 Gy challenge dose could be partly reversed by pre-treatment of cells with a dose of 0.5 Gy. (**a**) Representative flow cytometry dot plots of A549 cells after challenge with a dose of 2 Gy. At 24 h post-2 Gy IR, cells were harvested, stained with Annexin V/PI and analyzed using a flow cytometer. Apoptosis is shown as values expressed as fold change relative to control (Ctrl). (**b**) Bar graph depicting the percentage of membrane-intact and membrane-damaged cells following 2 Gy exposure, with and without a 0.5 Gy priming dose. (**c**) Reverse transcription-quantitative PCR results for the detection of HMOX1, SOD, Bcl2, IL-1β and TNF-α mRNA expression. Twenty-four hours after exposure to 0.5 Gy, A549 cells were harvested, and target genes expression relative to β-actin were determined by real-time PCR. Data are means ± SEM (*n* = 2); *P < 0.05, ***P < 0.001, ****P < 0.0001 by Student’s t test.

Indeed, previously published work on the response of A549 cells to LDIR is inconclusive. A 100-mGy priming dose has not been found to induce an adaptive response to a subsequent 6 h later challenge dose^22^. Increasing the interval between doses to 24 h did not produce different results^23^. However, raising the priming dose to 200 mGy was associated with protection against apoptotic death induced by a challenge dose of 20 Gy^24^. In our study, Cells pre-exposed to a 0.5 Gy “primed” dose before a 2 Gy exposure showed a lower percentage of apoptosis than those exposed to the 2 Gy alone (*P* < 0.05), indicating the presence of an adaptive response (Fig. 5a). Figure 5b show that ∼45% of cells treated with 0.5–2 Gy retained membrane integrity, whereas only 27% of those treated with the challenge dose alone did so. The molecular mechanisms of the hormesis phenotype involve antioxidant production and apoptosis pathways regulation, analogous to observations in normal cells. A 0.5 Gy pretreatment of lung adenocarcinoma cells significantly increased the mRNA expression of *HMOX1*, *SOD*, and *Bcl2*, which encodes intrinsic apoptosis pathway control protein. A549 cells also showed a marked reduction in the release of IL-1β (*P* < 0.05) and TNF-α (*P* < 0.01) following 0.5 Gy ionizing radiation exposure (Fig. 5c).

## Discussion

In this study we investigated the effects of exposing Lung adenocarcinoma A549 cells and normal lung fibroblasts WI-38 to ionizing radiation with or without administrating a 0.5 Gy priming dose. Evidence indicates that the adaptive response is not immediately expressed, it requires a time-lag. Consequently, Cells were primed 24 h before administration of the challenging dose. The 24-hour interval was chosen in accordance with previously published studies^25^. In WI-38, there was a statistically significant difference between mean normalized tail moments when comparing treatment with the challenge dose alone to treatment with 0.5 Gy prior to the challenge dose (*P* < 0.05). Moreover, pretreatment with 0.5 Gy reduced G1 phase cell cycle arrest and cell death—either through apoptosis or mitotic catastrophe—induced by the subsequent 2 Gy exposure. These results confirm previous studies that indicate the so-called “low dose”, from the radiation therapy view, can induce resistance in mammalian cells to subsequent high doses. Of these, Otsuka report, where he and colleagues used the comet assay to analyze DNA damage in the spleens of C57BL/6 N mice. They found that DNA damage induced by a 1.6 Gy challenge dose was significantly reduced in the mice that had been preirradiated at 1.2 mGy/h for 23 days (0.5 Gy in total) compared with that in the sham-irradiated mice^26^. A study on 30-day mortality found that mice pre-exposed to 0.5 Gy at a rate of 0.3 Gy/min had better survival rates than un-adapted mice after a subsequent exposure to 6.50 Gy of carbon ions (290 MeV/nucleon)^27^. Saini et al.^28^ investigated the expression levels of selected DNA damage response genes in G0 peripheral blood mononuclear cells (PBMCs) following irradiation. Blood samples from 25 healthy volunteers were exposed to 0.6 Gy priming dose followed by a challenging dose of 2 Gy, 4 h later. The transcription levels of ATM, ATR, MDM2, GADD45A, and CDKN1A genes were not altered, while Cyclin E and CDK2 expressions were higher in cells pre-exposed to a 0.6 Gy “adaptive” dose followed by the 2 Gy exposure than in those exposed to the 2 Gy dose alone.

Pre-exposing A549 cells to 0.5 Gy significantly reduced apoptosis induced by the subsequent 2 Gy dose, raising questions about previous findings on the differential responses of tumor and normal tissues to low-dose radiation^23^. Specifically, low-dose irradiation has been shown to protect normal lung epithelial cells from damage of a subsequent 5 Gy challenging dose, likely through the activation of the ATM/AKT/GSK-3β pathway. However, this protective effect was not seen in A549 cells under similar conditions.

The mechanism by which 0.5 Gy reduces vulnerability to subsequent ionizing radiation exposure is unclear. The study by Dieriks et al.^25^ aimed to investigate the adaptive response induced in human primary fibroblasts exposed to 0.5 Gy of X-ray 24 h before a 2 Gy dose. The 0.5 Gy priming altered the response of the DNA repair protein, phosphorylated histone H2AX (named γH2AX), and increased secreted cytokine levels relative to non-primed cells. Interestingly, these effects were not replicated by priming the cells with cytokines IL-6 nor TGF-β, indicating the adaptive response is regulated by another signaling pathways. Here, HMOX1 expression was elevated in primed cells, where HO-1, encoded by the HMOX1 gene, interacts with Nrf2 to increase the expression of various genes involved in the detoxification and elimination of oxidative stress, including SOD. This increase in antioxidant expression mitigated cellular damage from reactive oxygen species induced by high-dose radiation. Additionally, cells responded to a priming 0.5 Gy by increasing the expression of Bcl2 and decreasing of IL-1β and TNF-α. These changes may explain primed cells’ resistance to cell death induced by the subsequent 2 Gy dose.

Collectively, the 0.5 Gy priming help cells resist subsequent challenge dose and evade induced damages by modulating both HO-1/Nrf2 (antioxidant production) and apoptosis signaling pathways. Both normal and cancer cells have survived, posing a barrier to the clinical application of LDIR in cancer therapy.

## Data Availability

The datasets used and/or analyzed during the current study available from the corresponding author on reasonable request.
